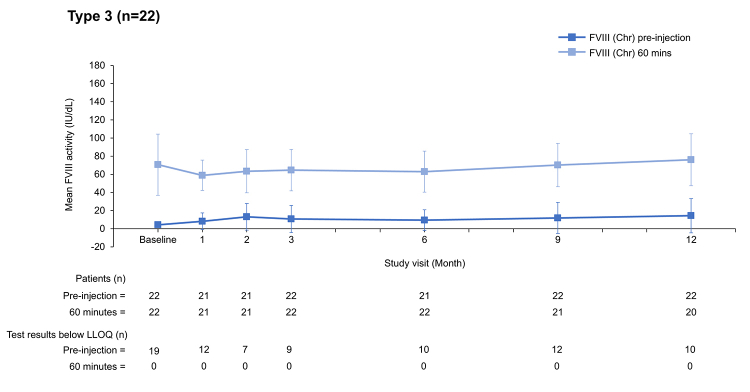# Factor VIII and von Willebrand factor activity levels during long-term prophylaxis with wilate—Analyses from the WIL-31 study

**DOI:** 10.1016/j.rpth.2025.103327

**Published:** 2025-12-30

**Authors:** Robert F. Sidonio, Ana Boban, Claudia Djambas Khayat, WIL-31 Study Investigators

**Affiliations:** 1 Department of Pediatrics, Emory University School of Medicine, Atlanta, Georgia, USA; 2 Haemophilia Centre, Department of Hematology, University Hospital Centre Zagreb, Zagreb, Croatia; 3 School of Medicine, University of Zagreb, Zagreb, Croatia; 4 Hotel Dieu de France Hospital, Saint Joseph University, Beirut, Lebanon

**Keywords:** factor VIII, prophylaxis, thrombosis, von Willebrand disease, von Willebrand factor

## Abstract

**Background:**

Long-term von Willebrand factor (VWF) prophylaxis is recommended for people with von Willebrand disease (VWD) who experience frequent and severe bleeds. However, repeated administration of VWF-containing concentrates may lead to VWF and/or factor (F)VIII accumulation, with an increased thrombotic risk. Data on FVIII and VWF accumulation during prophylaxis in VWD are lacking.

**Objectives:**

This study analyzed FVIII and VWF activity levels during long-term prophylaxis with wilate, a plasma-derived VWF/FVIII concentrate containing VWF and FVIII in a physiological 1:1 activity ratio, in the prospective WIL-31 study.

**Methods:**

Preinjection and postinjection FVIII and VWF activity levels were measured in plasma at baseline and after 1, 2, 3, 6, 9, and 12 months of wilate in the 33 patients who completed WIL-31. Analyses were descriptive and included stratification by age and VWD type.

**Results:**

VWF and FVIII activity levels (IU/dL) remained stable during 12 months of prophylaxis. Mean (SD) VWF activity levels preinjection and postinjection were 6.7 (4.0) and 59.0 (28.7) at baseline and 8.6 (7.6) and 44.4 (20.5) at 12 months, respectively. For FVIII, levels were 13.2 (18.9) and 75.5 (31.3) at baseline and 27.5 (25.6) and 83.6 (30.0) at 12 months, respectively. Similarly, when stratified by age and VWD type, no accumulation of either factor was observed. No thrombotic events were reported.

**Conclusions:**

During 12 months of wilate prophylaxis, there was no accumulation of FVIII or VWF regardless of age and VWD type, and no thrombotic events were reported. These findings from WIL-31 confirm and extend wilate’s existing safety data.

## Essentials

• High FVIII/VWF levels may increase the risk of thrombosis when treating von Willebrand disease.
• FVIII and VWF levels were measured during 12 months of wilate prophylaxis in the WIL-31 study.
• During WIL-31, there was no accumulation of FVIII or VWF, and no thrombotic events were reported.
• These WIL-31 data confirm and extend the existing body of safety data for wilate.

## Graphical abstract

**Figure:**
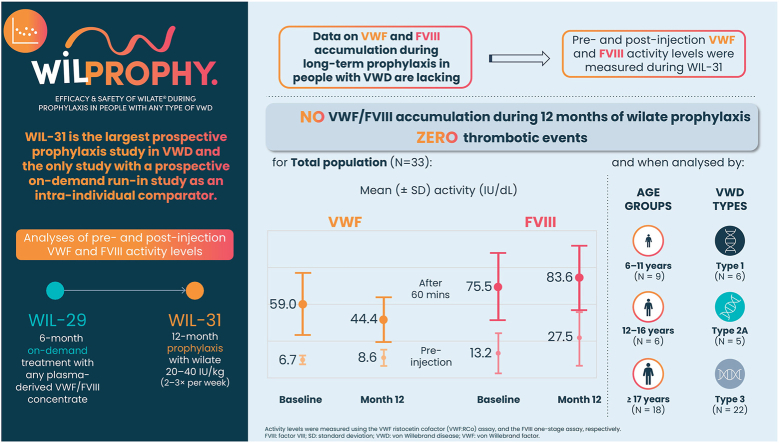

## 1 Introduction

Von Willebrand disease (VWD) is the most common inherited bleeding disorder [1,2] and is caused by quantitative (types 1 and 3) or qualitative (type 2) deficiencies of von Willebrand factor (VWF) [1,3]. A wide array of bleeding severity phenotypes exists among the different VWD types and subtypes [1]. Treatments for VWD include desmopressin and VWF replacement therapy, through use of VWF-containing concentrates (plasma-derived or recombinant) [3,4]. Plasma-derived concentrates also contain factor (F)VIII, although ratios of the 2 factors vary considerably between different products [3].

While therapy with VWF-containing concentrates aims to increase factor levels [3], highly elevated VWF and FVIII levels have both been associated with increased thrombotic risk [[5], [6], [7]]. In VWD, highly elevated factor levels may result from administration of repeated and frequent doses during surgical prophylaxis, which can result in transient yet marked elevations, or with long-term prophylaxis where repeated dosing could result in accumulation over time [8]. Thrombotic events are rare in patients with VWD and have been reported predominantly in surgical settings [9]. However, there is a lack of consensus on the levels of FVIII and VWF deemed to increase thrombotic risk—varying thresholds have been reported for patients with VWD undergoing surgery for FVIII (>250% [10,11] and >150% [4,12,13]) and VWF (>200% [11] to >150% [4]), with these thresholds being particularly important in patients at higher thrombotic risk [4].

International guidelines recommend long-term VWF prophylaxis for people with VWD who have frequent and severe bleeds [4]. However, published data on the levels of FVIII and VWF following long-term VWF prophylaxis are lacking. The WIL-31 study was the largest prospective clinical trial to investigate the efficacy and safety of VWF prophylaxis in patients with VWD [14]. Data from this study showed that prophylaxis with wilate, a plasma-derived VWF/FVIII (pdVWF/FVIII) concentrate containing VWF and FVIII in a physiological 1:1 activity ratio, is efficacious and well tolerated in adults, adolescents, and children with all VWD types [14]. This study reports FVIII and VWF activity levels during prophylaxis with wilate for 12 months in WIL-31.

## 2 Methods

The study design for WIL-31 (WİLPROPHY) has been published elsewhere [14]. In brief, WIL-31 was a prospective, noncontrolled, international phase 3 study, which investigated the efficacy and safety of wilate prophylaxis in patients with severe VWD. Of the 44 patients enrolled in WIL-31, 43 patients received wilate prophylaxis. Ten patients were excluded from the analyses due to unconfirmed VWD status (VWF ristocetin cofactor [VWF:RCo] >30 IU/dL), resulting in 33 patients for the analyses. To be eligible for inclusion in WIL-31, patients must have completed a prospective 6-month run-in study (WIL-29) with on-demand treatment with any available VWF concentrate.

During WIL-31, patients received prophylaxis with wilate for 12 months, with a recommended dose of 20 to 40 IU/kg bodyweight 2 to 3 times weekly. Patients aged ≥6 years at screening were eligible for inclusion in WIL-31. Patients were stratified by age into children (6-11 years), adolescents (12-16 years), and adults (≥17 years) and by VWD type into types 1, 2, and 3 [14].

### 2.1 FVIII and VWF activity levels over time

All patients who completed the WIL-31 study (*N* = 33) underwent factor-level assessments. Plasma FVIII and VWF activity levels were measured at baseline and at each study visit (after 1, 2, 3, 6, 9, and 12 months of treatment), using the VWF:RCo assay, and the FVIII 1-stage (OS) and chromogenic assays. Measurements were performed in all patients before and after administration of a prophylactic dose, except for the baseline measurement in children/adolescents where a fixed dose of 60 ± 10 IU/kg was administered. Predose and postdose FVIII and VWF activity levels were calculated from blood samples taken within 60 minutes before and 60 ± 5 minutes after wilate injection, respectively.

### 2.2 Statistical methods

Descriptive analyses were performed for the total population and stratified by age and by VWD type. Formal statistical analyses were not performed due to small sample size constraints. The lower limits of quantification for the FVIII OS and VWF:RCo assays were 3.1 and 5.0 IU/dL, respectively; if FVIII/VWF levels could not be detected by the corresponding assays, lower limits of quantification values were used as substitutes. Predose and postdose FVIII and VWF activity levels are shown as means ± SD.

### 2.3 Ethics/consent

The study was performed in compliance with the Declaration of Helsinki and the respective local regulations. Before initiating any study-related procedures, patients (or their legal guardians) provided voluntarily given, fully informed written and signed consent. Children old enough to understand the risks and benefits of the study were also informed and provided separately prepared assent forms [14].

## 3 Results and Discussion

The demographics and baseline characteristics of the 33 patients have been published [14]. In brief, the majority of patients (67%; *n* = 22) had type 3 VWD, with 18% (*n* = 6) having severe type 1 and 15% (*n* = 5) having type 2A. Nineteen patients (58%) were males. At screening, the median (range) age was 18 (7-61) years, and 55% (*n* = 18) patients were ≥17 years [14]. The median (range) weekly prophylactic wilate dose during WIL-31 was 58 (28-114) IU/kg [14], and median (range) prophylactic wilate dose per injection was 32 (20-45) IU/kg.

**Figure 1:**
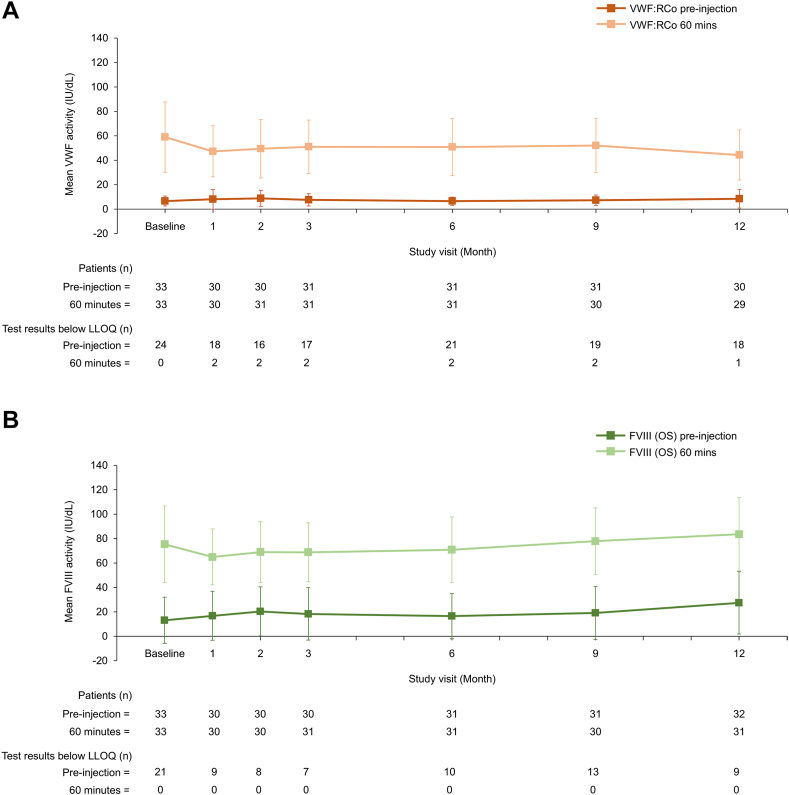
VWF and FVIII plasma activity levels at each study visit (*N* = 33). Data are mean ± SD. (A) VWF (VWF:RCo assay), (B) FVIII (1-stage assay). FVIII, factor VIII; LLOQ, lower limit of quantification; OS, 1-stage; VWF:RCo, VWF ristocetin cofactor; VWF, von Willebrand factor.

**Figure 2:**
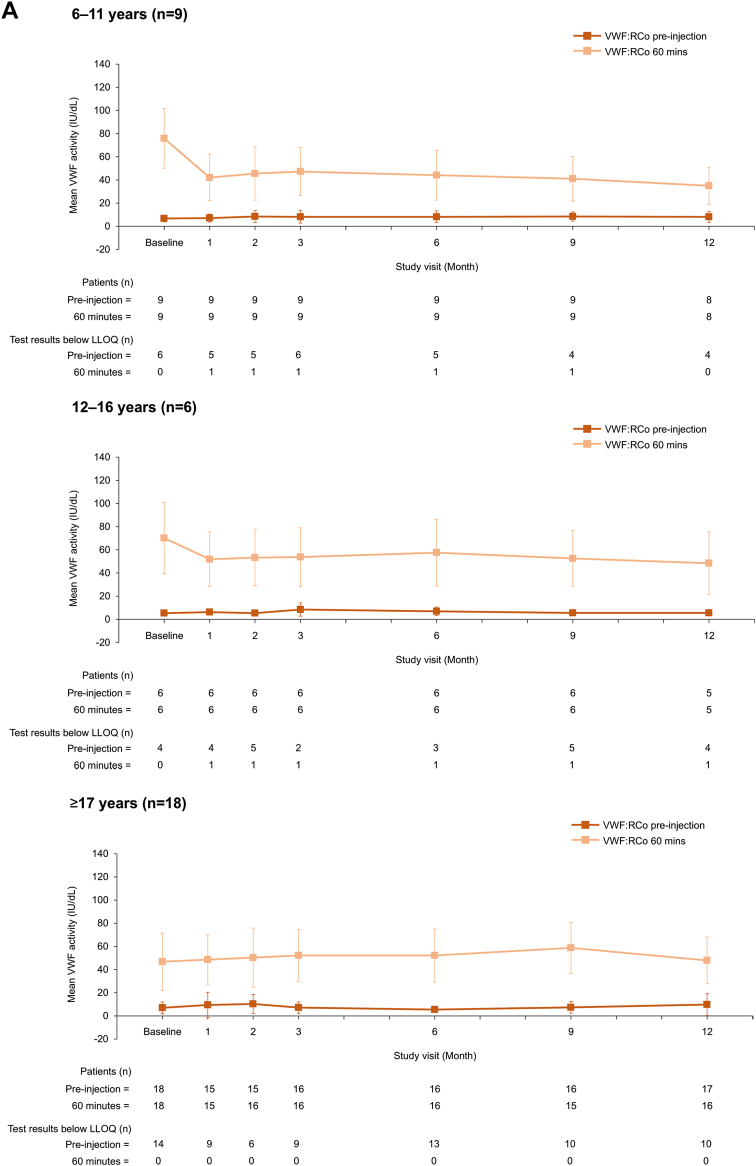

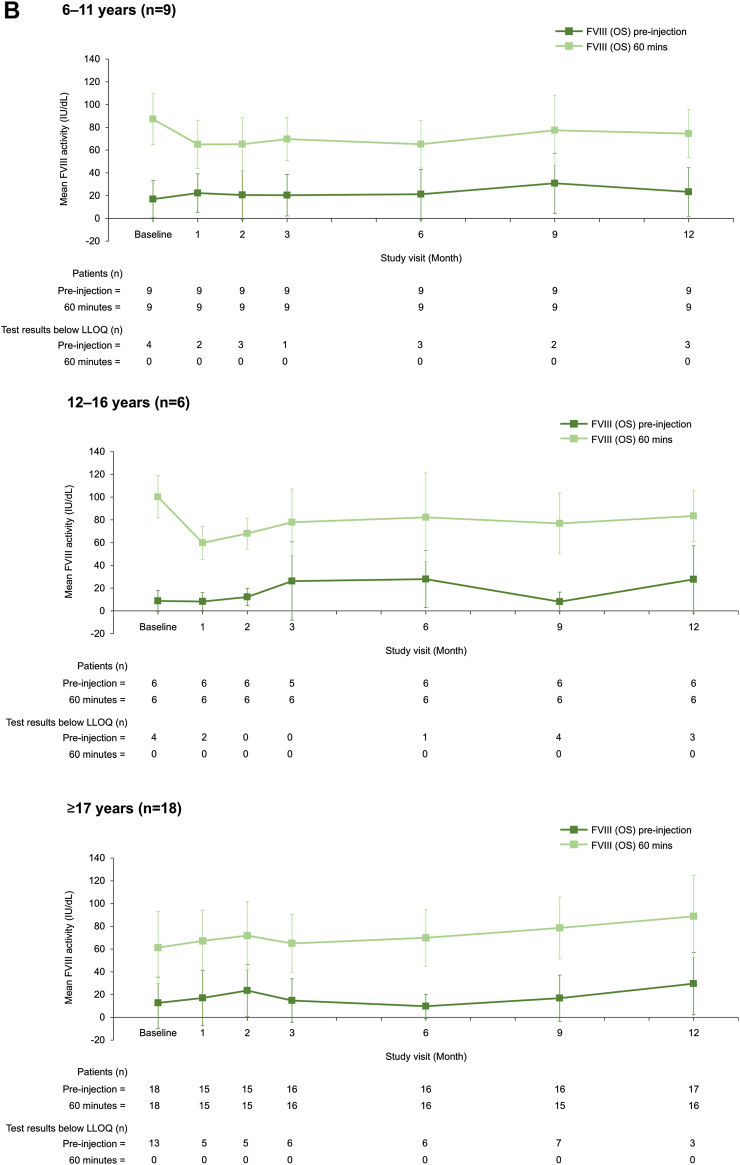
VWF and FVIII plasma activity levels at each study visit, stratified by age (*N* = 33). Data are mean ± SD. (A) VWF (VWF:RCo assay), (B) FVIII (1-stage assay). Data were stratified into 3 age groups: children (6-11 years), adolescents (12-16 years), and adults (≥17 years). FVIII, factor VIII; LLOQ, lower limit of quantification; OS, 1-stage; VWF:RCo, VWF ristocetin cofactor; VWF, von Willebrand factor.

**Figure 3:**
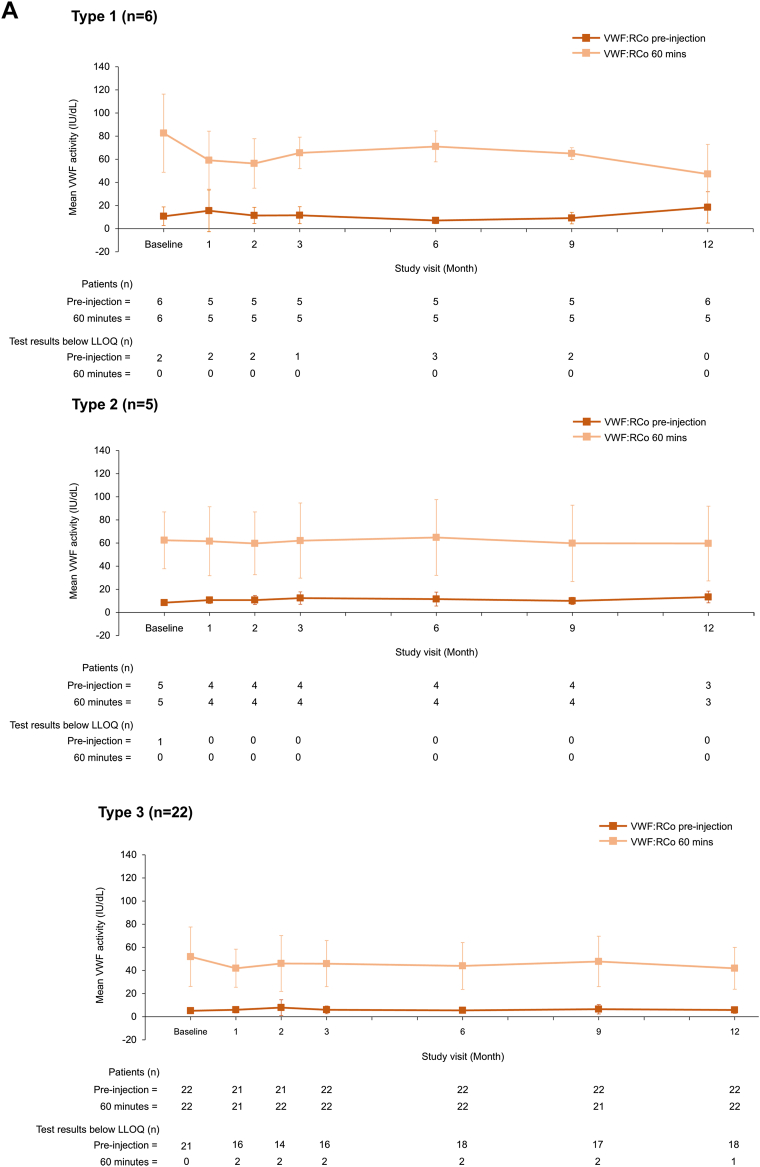

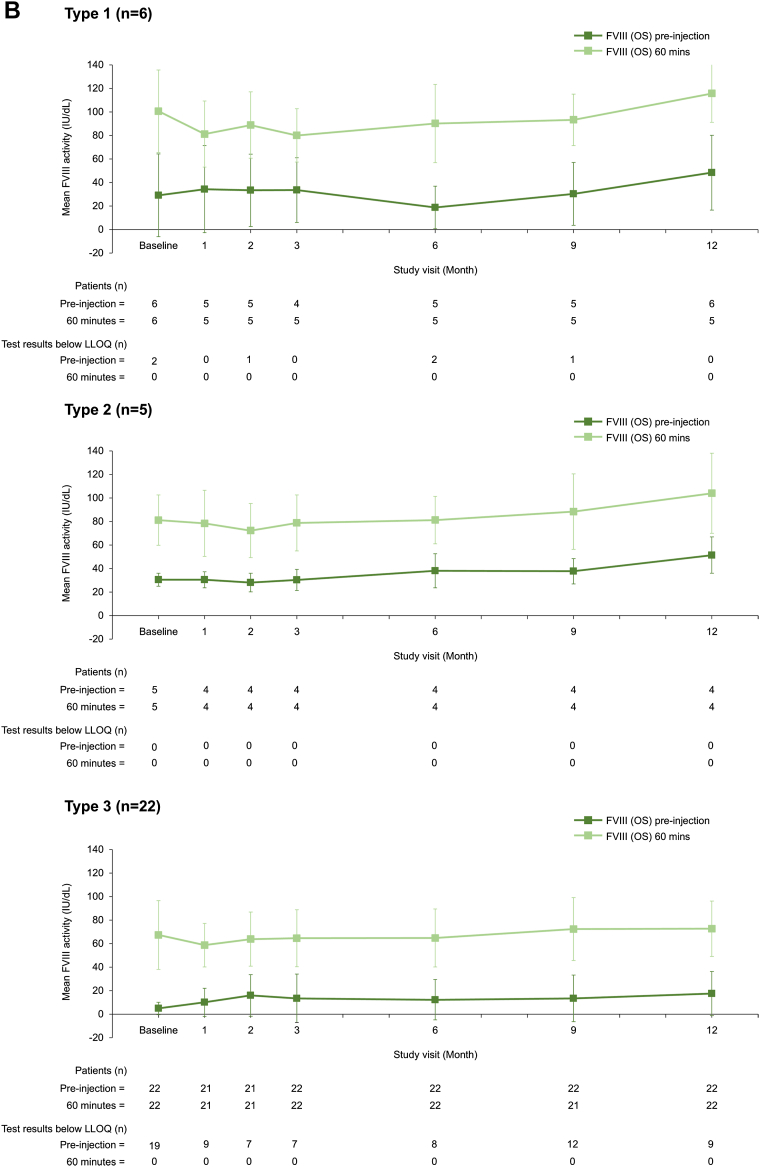
VWF and FVIII plasma activity levels at each study visit, stratified by VWD type (*N* = 33). Data are mean ± SD. (A) VWF (VWF:RCo assay), (B) FVIII (1-stage assay). Data were stratified into VWD types: severe type 1, type 2, and type 3. FVIII, factor VIII; LLOQ, lower limit of quantification; OS, 1-stage; VWF:RCo, VWF ristocetin cofactor; VWF, von Willebrand factor.

**Supplementary Figure 1:**
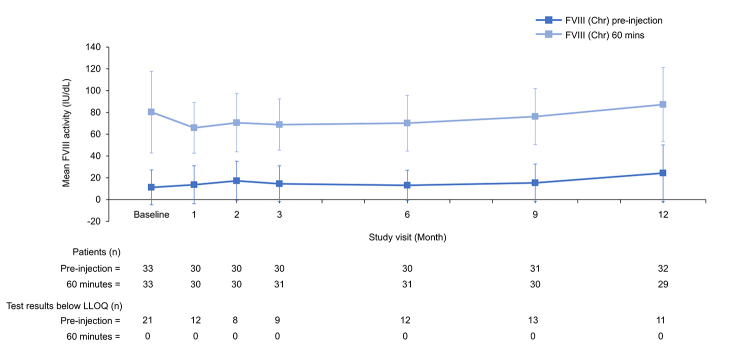


To our knowledge, this is the first published report of VWF/FVIII levels during long-term prophylaxis with a VWF-containing concentrate. VWF and FVIII activity levels remained stable during the 12-month prophylaxis period with no accumulation of either factor (Figure 1), regardless of age and VWD type (Figures 2 and 3). Mean (SD) VWF activity levels (IU/dL) preinjection and postinjection were 6.7 (4.0) and 59.0 (28.7) at baseline and 8.6 (7.6) and 44.4 (20.5) at 12 months, respectively (Figure 1). For FVIII (OS assay), mean preinjection and postinjection activity levels (IU/dL) were 13.2 (18.9) and 75.5 (31.3) at baseline and 27.5 (25.6) and 83.6 (30.0) at 12 months, respectively (Figure 1). No FVIII accumulation was also observed when using the chromogenic assay (Supplementary Figures 1–3). When stratified by VWD type, preinjection and postinjection VWF and FVIII activity levels appeared slightly lower for patients with type 3 VWD than those for patients with type 1 and type 2 VWD (Figure 3). This is unsurprising given the undetectable plasma levels of VWF and very low FVIII plasma levels in patients with type 3 VWD [3].

In VWD, the aim of therapy is to correct the dual haemostatic defects of abnormal platelet adhesion-aggregation due to a partial or complete lack of VWF, and intrinsic coagulation due to low FVIII levels [3]. While increased factor levels are thus desirable in the treatment of VWD, avoidance of markedly elevated factor levels is recommended to reduce potential thromboembolic complications [4,[10], [11], [12], [13]]. During WIL-31, none of the 33 patients experienced levels elevated above the published thresholds of 250% for FVIII and 200% for VWF during 12 months of wilate prophylaxis (Table). Using the lower threshold of 150%, no patients developed VWF levels above this threshold, and only 3 patients experienced FVIII levels >150% (Table).

**Table:** Postinjection FVIII and VWF activity levels at each study visit.

Study visit	*n*	Mean	SD	Median	Q1	Q3	Minimum	Maximum
FVIII activity (OS assay; IU/dL)								
Baseline	33	75.5	31.3	76.0	58.2	89.0	22.4	151.6^a^
Month 1	30	65.0	22.9	56.8	46.9	73.6	39.2	126.1
Month 2	30	69.1	24.8	64.3	51.7	86.2	32.5	129.0
Month 3	31	68.9	24.2	63.9	51.6	88.6	29.8	132.7
Month 6	31	71.0	26.8	63.5	50.3	82.2	31.1	139.9
Month 9	30	78.0	27.2	74.8	60.7	94.1	33.0	133.3
Month 12	31	83.6	30.0	75.7	61.1	102.8	40.3	158.4
FVIII activity (Chr assay; IU/dL)								
Baseline	33	80.3	37.5	76.2	54.2	101.5	13.8	182.0^a^
Month 1	30	65.9	23.3	57.8	50.0	77.2	33.4	131.4
Month 2	30	70.6	26.7	69.4	51.2	81.1	27.2	133.4
Month 3	31	68.9	23.5	65.1	52.6	80.5	32.1	125.5
Month 6	31	70.1	25.6	68.6	51.9	77.6	26.9	127.7
Month 9	30	76.2	25.8	74.5	59.4	87.6	34.3	135.5
Month 12	29	87.3	34.0	76.1	63.9	108.8	38.1	171.1^a^
VWF:RCo (IU/dL)								
Baseline	33	59.0	28.7	55.9	35.8	80.7	9.2	128.1
Month 1	30	47.4	21.0	42.5	36.0	61.5	5.0	101.6
Month 2	31	49.5	23.9	48.0	30.9	72.0	5.0	95.8
Month 3	31	51.1	21.9	53.0	35.9	60.9	5.0	108.8
Month 6	31	50.9	23.4	49.1	35.5	66.1	5.0	110.0
Month 9	30	52.2	22.3	58.5	37.6	63.8	5.0	106.9
Month 12	29	44.4	20.5	42.7	30.7	58.1	5.0	94.4

Chr, chromogenic; FVIII, factor VIII; IU, international unit; OS, 1-stage; Q1, lower quartile; Q3, upper quartile; VWF:RCo, VWF ristocetin cofactor; VWF, von Willebrand factor.

a Three patients experienced FVIII levels >150%. Patient 1 had levels at baseline of 151.6% (OS) and 182.0% (Chr); patient 2 had levels at 12 months of 158.4% (OS) and 150.0% (Chr); patient 3 had levels at 12 months of 171.1% (Chr; levels measured by the OS assay [148.4%] were below the threshold).

Despite the potential increased risk for thrombotic events with high factor levels, reports of thrombotic complications from studies of VWF-containing concentrates are rare [1]. Most cases have been reported in patients with other underlying risk factors for thrombotic events (eg, older age, obesity, and surgery) and/or who were not receiving thromboprophylaxis [2,8,15]. Due to the lack of publications relating to long-term prophylaxis and factor accumulation, we draw on the literature published for surgery where high factor activity levels and/or the occurrence of thrombotic events have been described for a number of other VWF-containing concentrates. An analysis of 2 studies of a pdVWF/FVIII concentrate with a VWF:FVIII ratio of 2.4:1 (Humate-P/Haemate P; CSL Behring) found transient elevations in VWF and/or FVIII levels (≥150%) during surgery in most patients [16]. In the first study, despite these elevations and modest accumulation of FVIII in some patients, no thromboembolic events were reported [17]. In the second study, preinfusion FVIII activity levels increased progressively over time [18]. One patient developed thrombophlebitis [18], while another experienced a pulmonary embolism [18], with FVIII and VWF activity levels reaching ≥300% and ≥200%, respectively, in both patients [16]. Marked elevations of both VWF and FVIII, and FVIII accumulation over time, were also observed in a retrospective cohort study of surgical patients receiving multiple doses of Humate-P; however, there were no thrombotic complications [19].

In a study of another VWF/FVIII concentrate (Alphanate; Alpha Therapeutic) used prophylactically in a surgical setting, 2 patients experienced thrombotic events—arm phlebitis at the injection site and deep vein thrombosis (DVT) postsurgery [20]. Although the DVT was reported to may be related to increased postoperative FVIII levels, the patient also had poor postoperative mobilization due to a prior knee arthrodesis [20]. Furthermore, the ratio of VWF:FVIII is known to vary between different lots of Alphanate (US Prescribing Information), with the ratio ranging from 0.4 to 1.0 in the lots used in the reported study [20].

Thrombotic events have also been reported with vonicog alfa (Vonvendi; Takeda) [21,22], a recombinant VWF concentrate practically devoid of FVIII [23]. In a surgical setting, 1 patient undergoing a hip replacement developed proximal DVT postoperatively, which was deemed to be possibly related to treatment [21]. However, the patient also had numerous risk factors for thrombosis including obesity, surgical procedure, and immobilization [21]. When Vonvendi was used for prophylaxis over 12 months, 1 of the 23 patients experienced a thromboembolic event, although it was not deemed DVT (a case of nonserious, nonsevere purpura that resolved without action) [22].

Our findings support a growing body of data for wilate demonstrating no factor accumulation and no thrombotic events in clinical trials and real-world studies [[24], [25], [26], [27], [28]]. In an open-label, multinational, phase 3 trial of wilate for surgical prophylaxis in 28 patients with VWD for ≥6 years, there was no FVIII or VWF accumulation after repeat dosing [25]. Despite 6 patients experiencing FVIII activity levels of >250 IU/dL during maintenance infusions, which mostly correlated with higher doses and were corrected by reducing the dose, no thromboembolic events occurred during the postoperative follow-up period [25]. A single-center cohort study in 125 patients with VWD receiving repeated wilate infusions for surgical prophylaxis reported no accumulation of FVIII or VWF in those patients for whom factor activity levels were measured over time (up to 120 hours) [24]. In this study, no thromboembolic events occurred during the follow-up of up to 4 weeks after surgery [24]. Although high peak FVIII levels were observed in 20.8% patients in the first 48 hours postinjection, these were transient and mostly related to limited *in vivo* recovery measurements of FVIII activity with wilate, and factors such as pregnancy and obesity, with dosing in these cases being primarily based on VWF rather than FVIII levels [24].

Wilate contains VWF and FVIII in a physiological 1:1 activity ratio [29], closely resembling the ratio found in normal human plasma [30]. This 1:1 ratio may simplify dosing and monitoring [29], and allows correction of both VWF and FVIII levels [23]. pdVWF/FVIII products with higher levels of VWF mostly contain up to 10 times as much VWF relative to FVIII, with one product considered to be almost devoid of FVIII [23]. Furthermore, the recombinant VWF product Vonvendi has VWF levels over 100 times greater than FVIII [23]. While it might be assumed that products containing less FVIII than VWF would be preferable in terms of avoiding FVIII accumulation, the amount of unbound VWF in the plasma has been reported to influence FVIII accumulation, with higher plasma levels of VWF associated with an increased half-life for FVIII (endogenous production of which is not impaired in VWD) and decreased clearance for FVIII [31].

Current guidelines recognize the need for more prospective clinical trials for use of prophylaxis in VWD [4]. The WIL-31 study has contributed toward addressing this unmet need with its findings leading to the updated indication in the US Prescribing Information to include prophylaxis in VWD, with its current indications in VWD including treatment of bleeds, perioperative management of bleeding, and prophylaxis, in children and adults with any VWD type in both Europe (wilate Summary of Product Characteristics) and the US (wilate US Prescribing Information).

Strengths of the analyses were that data included a diverse population of all age groups and all VWD types and that WIL-31 provides long-term data on FVIII and VWF activity. Limitations include that the majority of patients (97%) were Caucasian, and no patients had type 2N VWD [14]. Due to the small patient numbers in the stratified analyses, these analyses were descriptive only and no formal statistical testing was performed. Although evaluating FVIII and VWF levels according to dosing regimen would be of interest, this was not explored in this report because only 3 patients received 3 times weekly dosing throughout the study. However, a separate article describing 7 patients who increased dosing frequency from 2 times weekly to 3 times weekly during the study showed that factor levels remained stable following the dose frequency increase [32].

## 4 Conclusions

These WIL-31 data confirm wilate’s existing body of safety data. There was no accumulation of FVIII or VWF, and no thrombotic events were reported, in 33 patients during 12-months of wilate prophylaxis, regardless of age and VWD type.

## Figures and Tables

**Supplementary Figure 2A:**
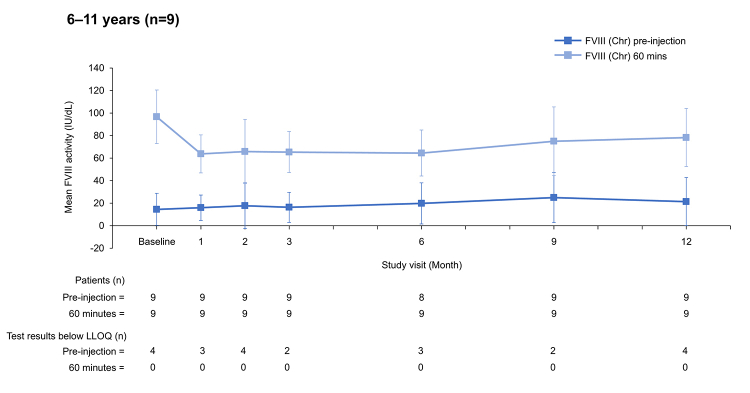


**Supplementary Figure 2B:**
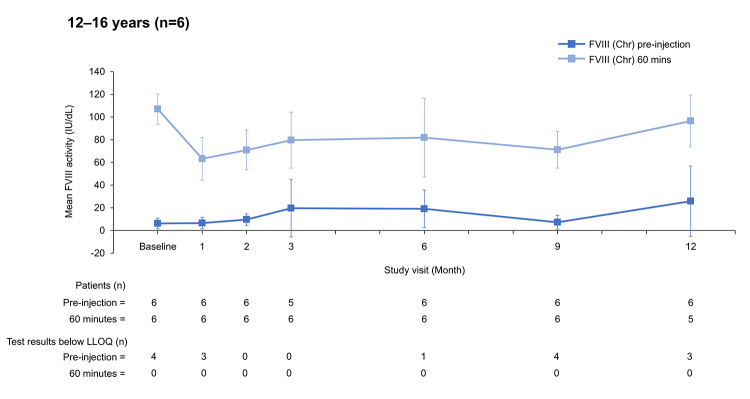


**Supplementary Figure 2C:**
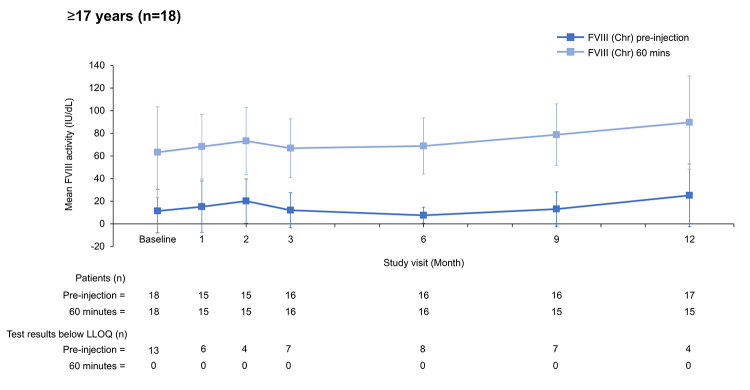


**Supplementary Figure 3A:**
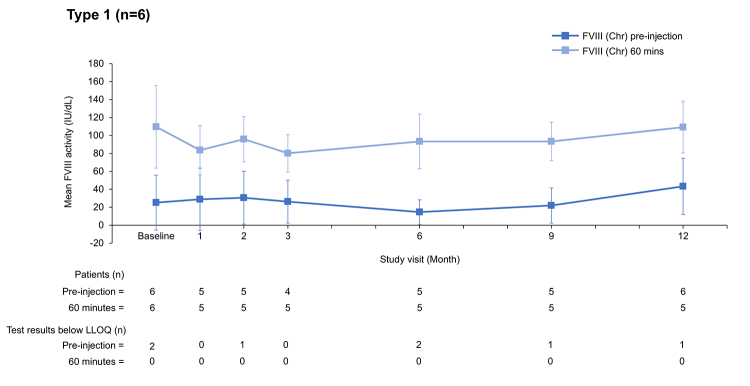


**Supplementary Figure 3B:**
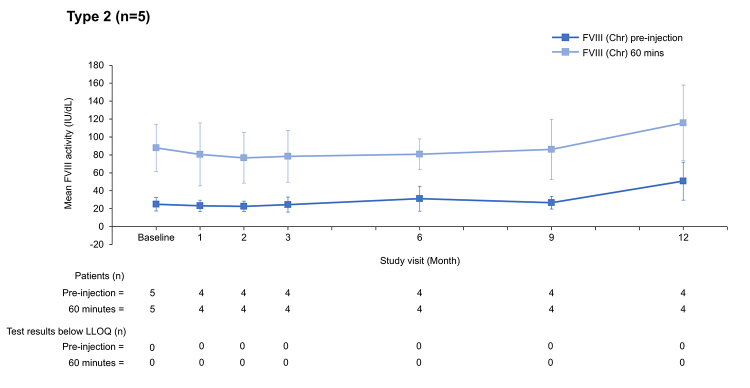


**Supplementary Figure 3C:**